# Market integration, income inequality, and kinship system among the Mosuo of China

**DOI:** 10.1017/ehs.2022.52

**Published:** 2022-11-21

**Authors:** Siobhán M. Mattison, Neil MacLaren, Chun-Yi Sum, Peter M. Mattison, Ruizhe Liu, Mary K. Shenk, Tami Blumenfield, Mingjie Su, Hui Li, Katherine Wander

**Affiliations:** 1 University of New Mexico, Department of Anthropology, Albuquerque, NM, USA; 2 State University of New York at Buffalo, Department of Mathematics, Buffalo, NY, USA; 3 Boston University, Department of General Studies, Boston, MA, USA; 4 University of New Mexico, Department of Biology, Albuquerque, NM, USA; 5 Pennsylvania State University, Department of Anthropology, University Park, PA, USA; 6 Yunnan University, School of Ethnology and Sociology, Kunming, Yunnan, China; 7 Fudan University, MOE Key Laboratory of Contemporary Anthropology, Shanghai, China; 8 Binghamton University (SUNY), Department of Anthropology, Binghamton, NY, USA

**Keywords:** Matriliny, patriliny, economic development, material wealth, communal institutions

## Abstract

Increased access to defensible material wealth is hypothesised to escalate inequality. Market integration, which creates novel opportunities in cash economies, provides a means of testing this hypothesis. Using demographic data collected from 505 households among the matrilineal and patrilineal Mosuo in 2017, we test whether market integration is associated with increased material wealth, whether increased material wealth is associated with wealth inequality, and whether being in a matrilineal vs. patrilineal kinship system alters the relationship between wealth and inequality. We find evidence that market integration, measured as distance to the nearest source of tourism and primary source of household income, is associated with increased household income and ‘modern’ asset value. Both village-level market integration and mean asset value were associated negatively, rather than positively, with inequality, contrary to predictions. Finally, income, modern wealth and inequality were higher in matrilineal communities that were located closer to the centre of tourism and where tourism has long provided a relatively stable source of income. However, we also observed exacerbated inequality with increasing farm animal value in patriliny. We conclude that the forces affecting wealth and inequality depend on local context and that the importance of local institutions is obscured by aggregate statistics drawn from modern nation states.

## Graphical abstract

**Figure:**
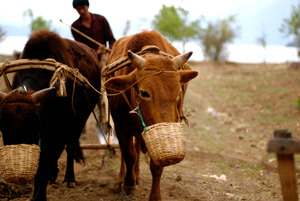

**Social media summary:** Is patriarchy prone to inequality?

## Introduction

Social inequality is a pervasive feature of human societies, but widespread, institutionalised inequality is considered evolutionarily novel. Indeed, humans are thought to have spent over 90% of their existence as relatively egalitarian hunter–gatherers, with persistent, institutionalised inequality emerging around the Holocene transition some 10,000–12,000 years ago (Mattison, Smith et al., 2016; but see Singh & Glowacki, 2022). Numerous factors are thought to have allowed for the initial evolution of persistent, institutionalised inequality. Ecological and archaeological evidence link increasing population density or pressure (e.g. Cohen 1977; Keeley 1988), advancing technology (e.g. Arnold 1991; Johnson & Earle 2000), and climate stability (e.g. Richerson et al., 2001) with increased inequality, for example. Agriculture is typically associated with increased inequality, prompting many (e.g. Kohler et al., 2017), although not all (Gurven et al., 2010), scholars to consider agriculture as a primary driver of inequality. Following Borgerhoff Mulder et al. (2009), we (Mattison, Smith et al., 2016) have argued that this broad array of causes may be consolidated by two key features: economic defensibility and the intergenerational transmission of wealth. In this paper, we investigate whether, as anticipated by this model, an increased emphasis on material wealth is associated with increased inequality. We further investigate how different gender biases that attend differences in norms of inheritance, descent and post-marital residence (‘kinship systems’) contribute to this association. The gender norms that arise in association with female- vs. male-biased kinship systems provide an important, but underused perspective on gendered conflict by linking gender biases to variation in production systems and downstream consequences affecting household and societal welfare.

We focus here on inequality in material wealth as it is especially conducive to inequalities based on defensibility, monopolisation and intergenerational wealth transmission. Material wealth is one of numerous forms of ‘wealth’ – ‘an attribute of the individual that contributes to a flow of valued goods and services’ (Bowles et al., 2010) – upon which humans rely to subsist and reproduce (Ziker et al., 2016). Embodied capital combines somatic capital (Kaplan et al., 2003) – energy captured from the environment and stored in the soma – and noetic wealth – education, skills and knowledge – to depict wealth that is stored in an individual. Social or relational capital is wealth that arises via relationships with others. Compared with these forms of wealth, material wealth – capital assets, private land, money – seems to play an outsized role in predicting the degree of inequality as measured by standard indices (see Tucker et al., 2015). Although moderate inequalities in embodied and social capital have been documented in hunting and gathering communities (e.g. Smith, Hill et al., 2010), the levels of inequality in material wealth far exceed these more moderate inequalities (e.g. Smith, Borgerhoff Mulder et al., 2010). Indeed, archaeological evidence such as burial sites suggests that there have long been lineage-based differences in the ownership of material resources (e.g. Kennett et al., 2017). Families can also differentially control and transmit other resources that are key determinants of reproductive success, including information, social capital and ‘embodied’ differences (Kaplan 1996) in health. As we (Mattison, Smith et al., 2016) and others (Borgerhoff Mulder et al., 2009; Shenk et al., 2010; Shennan, 2011) have previously argued, however, we believe that material wealth, all else equal, is especially conducive to inequality because it is both easily defended and transmitted to offspring, such that its differentials – arising from disparities in skills in acquisition or quasi-random processes (Smith et al., in press) – may be maintained across generations.

Although the hypothesis that material wealth exacerbates inequality was proposed long ago (e.g. Kuznets, 1955; see also Wu, 2019 for review), evidence for the hypothesis is mixed, and exactly how material wealth is expected to structure inequality remains unclear. Influential economic models of contemporary large-scale societies (Piketty, 2015; Piketty & Saez, 2014) have shown that asset-based wealth is subject to steeper inequalities than income. This is in part because assets grow exponentially and each increment of growth drives a deeper wedge between those with extensive assets and those without, especially during periods of low economic growth (Gudeman, 2015). This suggests that inequality has the potential to increase more sharply with increasing material wealth, at least in the context of capital markets. The so-called ‘Kuznets curve’ offers a more nuanced relationship between wealth and inequality, anticipating an inverted U shape as inequality initially increases but then decreases with wealth as the bottom of the wealth distribution falls out owing to a reduction in extreme poverty (Kuznets, 1955) or owing to some lag in time between the introduction of market forces and their effects on increasing incomes (Mao et al., 2022).

Market integration and economic development offer opportunities for depicting both general features of hypothesised relationships between wealth and inequality and the norms and institutions that may modify these relationships. These processes are altering lifeways in small-scale communities the world over in ways that may reinforce existing, or expose new, inequalities (Godoy, 2001; Haagsma & Mouche, 2013; Walker et al., 2016). Although difficult to capture using single metrics (Mattison, Hare et al., 2021), market integration as a general process involves transitions from subsistence activities to ones based more on participation in markets (Lu, 2007). As market integration takes hold, individuals, households and communities participate variably (Tucker et al., 2015). Decisions to participate depend on how characteristics at each level alter the costs and benefits of participation in market activities relative to maintaining subsistence-based lifeways (Demps & Winterhalder, 2019; Mattison, Hare et al., 2021). For example, individuals who are, early adopters, highly educated or have little to lose may be more willing to avail themselves of new opportunities presented by market integration (e.g. Gurven et al., 2015; also see Rogers 2010). Communities that are positioned closer to market activities may experience fewer opportunity costs of participating in markets (e.g. Gurven et al., 2015; Henrich, 1997). Household-level contexts may also affect market integration as individuals who are positioned in relatively educated households (even if they themselves are not educated) may experience market integration differently than those in different household contexts. Material wealth obviously increases in the context of previously autarkic (i.e. self-reliant) societies who, prior to market transition, have limited material wealth (e.g. Cashdan, 1980). Small-scale agricultural societies are already heavily reliant on material wealth compared with foragers and horticulturalists and correspondingly display some of the highest levels of inequality in comparative studies (Shenk et al., 2010). Still, market integration offers opportunities to accumulate new forms of wealth in cash economies, plausibly increasing the scope for furthering wealth-based inequalities. Here, we investigate whether indicators of market integration and village-level material wealth are associated with increasing inequality in an agricultural setting that is increasingly integrated into regional, national and international markets.

Finally, the potential for market integration and material wealth to create different or conflicting incentives by gender is theoretically plausible but under-researched. Here, we investigate the extent to which differences in prevailing gendered kinship norms modify any relationship between material wealth/market integration and inequality. Evolutionary theory provides a basis for expecting that matriliny – where inheritance and descent are biased in favour of daughters – should be less prone to steep inequalities than patriliny (Holden et al., 2003; Holden & Mace, 2003). Specifically, features of the ecologies thought to produce matriliny may minimise intergenerational inequality compared with those more conducive to patriliny because such ecologies limit the scope for men to advance reproductive and social agendas that drive inequality. Relatively expansive landscapes, in which land is not circumscribed (e.g. Divale, 1974), as well as offshore fishing and horticulture (Alesina et al., 2013; BenYishay et al., 2017; Douglas, 1969; Ember & Ember, 1971; Schneider, 1961), are thought to produce matrilinineal inheritance and descent whereas intensive agriculture creates situations in which parents who bias inheritance towards sons have higher reproductive success. This is because forms of wealth that are circumscribed or create economies of scale can create steeper reproductive returns via men (because men have high caps on reproduction and limited investment in each child; but see Brown et al., 2009). To put it another way, male reproductive agendas may diverge less from female reproductive agendas under matriliny, because the sources of wealth thought to produce matrilineal inheritance and descent are not disproportionately easy for men to translate into reproductive success compared to women (Alesina et al., 2013; Borgerhoff Mulder & Ross, 2019; Holden et al., 2003; Mattison, Quinlan et al., 2019). Furthermore, gendered norms and institutions that codify daughter- or son-biased inheritance and investment may lag behind economic circumstances thought to drive changes in gendered social systems. Thus, gendered kinship institutions – which are rooted in ecological differences that lead to lesser accumulation of defensible resources in matriliny (Holden et al., 2003; Mattison, 2011) – are likely both to reflect and to affect the dynamics of gendered conflict in ways that extend beyond interpersonal dynamics (Borgerhoff Mulder & Rauch, 2009; Gibson & Lawson, 2015; Lawson & Uggla, 2014). Here, we ask whether the relationship between emphasis on material wealth (market integration) and inequality differs according to the prevailing kinship system, hypothesising that matriliny buffers against escalating inequality compared to patriliny.

In sum, we test the following hypotheses:

1. Market integration, proxied by distance to the main regional centre of tourism, increases the emphasis on material wealth, proxied by income and various assets.
2. Increased emphasis on material wealth is associated with higher levels of inequality.
3. Kinship system mediates the above relationship such that the relationship is stronger in patrilineal communities compared with matrilineal ones.

## Population and socioeconomic context

We test these hypotheses using data collected in 2017 in Mosuo communities in southwestern China. The Mosuo are a Tibeto-Burmese people residing in the foothills of the Himalayas (Shih, 2010; Walsh, 2004). The Yongning basin, where flatlands can be used for rice cultivation, is the traditional home of the matrilineal Mosuo. Today's Mosuo practice mixed economies (see BurnSilver et al., 2016). Many people residing in the basin, which is closest to the historical regional market in Yongning town, retain traditional matrilineal practices and a family structure characterised by large matrilineal households where adult women and men live with their siblings and maternal kin. Most of these matrilineal households comprise at least six members; large families with 10 or 12 members or more are not uncommon (Mattison, Sum et al., 2021). Many households are headed by a senior woman in the family, who is responsible for managing domestic affairs and providing for the family. Everyone in the household – women and men, adults and children – helps in the rice paddy fields, especially during harvesting seasons. Neighbours from other households and villages, many of whom are genetically or affinally related, often assist one another when agricultural work is the busiest (Thomas et al., 2018). In addition to rice cultivation, almost all households keep livestock and fowl, including pigs, oxen, water buffalo, cattle, chickens and sometimes horses and goats. The quantity of livestock owned, pigs in particular, has traditionally been considered an indicator of household wealth (Shih, 2010).

The patrilineal Mosuo we visited reside in the more rugged mountains to the west of Yongning, centred around Labo Township (Harrell, 2001; Mathieu, 2003; Mattison, Beheim et al., 2016; Mattison, Sum et al., 2021; Shih, 1993). The scarcity of flat land in these areas is associated with very different family structures. Most households in this mountainous region are smaller and are nuclear or patrilineal-stem in structure. Some matrilineal values of gender egalitarianism and family-centred culture persist (Mathieu, 2003; Mattison, Sum et al., 2021), but it is not as common in this region to find large multigenerational matrilineal households where adult siblings co-reside. The average size of households in this patrilineal area is five to six people, consisting of an adult man with his wife, his children and sometimes his parents. In these patrilineally structured households, a man usually serves as the head of household. Another consequence of the rugged landscape is that many patrilineal Mosuo villages are situated far from each other, which makes intervillage visits challenging. Since horticulture in the mountains is less labour intensive than rice cultivation, interhousehold cooperation is less common (see Mattison, MacLaren et al., 2021). Finally, agriculturalists in this area have long relied on different crops, such as tobacco, and different livestock: oxen and water buffalos are uncommon given the steeper terrain, and feed for pigs more difficult and expensive to obtain. Sheep and goats are comparatively common.

Since the 1990s, tourism and migration have brought about substantial changes in the modes of subsistence and economic activities in Mosuo villages (Luo, 2008; Mattison, 2010; Walsh, 2005). As the scenic alpine lake adjacent to the Yongning basin developed into a major tourist attraction in China, the matrilineal region became increasingly reliant on a cash economy. In some matrilineal villages frequented by tourists, many Mosuo households have shifted away from agriculture and have converted parts of their compounds into guesthouses and hotels. Substantial portions of their farmland have been reclaimed by the government for road construction, village beautification or other tourism-related purposes, further decreasing the reliance on agriculture as a primary means of subsistence. Increases in the cost of living have also made subsistence farming less economically viable as a sole endeavour.

Tourism is not as developed in mountainous patrilineal villages, but its impact on the local economy is nonetheless considerable. Patrilineal Mosuo are not immune to tourism-related inflation in Yongning Township, where they usually shop for foodstuffs, fodder, clothing and agricultural necessities. Demand for cleaning and kitchen staff, tour guides, drivers and other hospitality providers creates attractive job opportunities for young Mosuo. Many move to nearby tourist towns and cities to work or start their own businesses, and patrilineal village rely increasingly on cash remittances and nascent local tourism endeavours. In both cases, labour migration has long been a common source of income, as have various government initiatives to alleviate poverty and decrease reliance on agriculture as a primary means of subsistence (Blumenfield et al., 2018).

Both matrilineal and patrilineal Mosuo villages are thus experiencing market integration, although in slightly different forms and at different paces. This paper examines the impact of these economic changes on reshaping the patterns of inequality in the region. To foreshadow the results and conclusions, we emphasise here that the differences in subsistence between matrilineal and patrilineal communities are stronger than we initially anticipated. As the results will show, patrilineal communities remain heavily reliant on agriculture and are removed enough from the tourism market that distance to the lake area does not seem to capture the forces driving market integration and related dynamics in the patrilineal area in ways that we anticipated.

## Methods

This paper drew from a sociodemographic survey conducted with 505 households over seven months in 2017. Fifteen Mosuo villages – including six matrilineal villages and nine smaller patrilineal villages – were surveyed. Eighteen households were located outside of the survey area and data from one household was not usable owing to a recording error, yielding usable data from 486 households. CYS, accompanied by a local research assistant, travelled house-to-house in these villages and invited a primary adult respondent in each house to supply information on household composition and wealth. We also asked the respondent to provide sociodemographic information on behalf of all members of the household, including their marital status, reproductive history, occupation, income and educational attainment. Each interview lasted approximate 30–90 min. Interviews were primarily conducted in Mandarin Chinese, and the research assistant occasionally translated responses from Naru (the Mosuo language) and the local Chinese dialect.

University of New Mexico's Institutional Review Board provided ethical oversight for the associated data collection (06915) with additional ethical review by Fudan University (16268).

### Wealth indices

The surveys above enumerated components of material wealth ranging from monthly income to various assets including household appliances and livestock for 486 households. We delegated a subset of assets to represent wealth in either farm animals (livestock and fowl) or ‘modern’ categories for each household. Farm animal worth included pigs, poultry, Caprinae (goats and sheep), Bovidae (cattle and water buffalo) and Equidae (horses, ponies, donkeys and mules). Modern assets included cars, motorcycles, flush toilets, refrigerators, air conditioners, washing machines, televisions, smart phones and computers. For each household, we calculated the monetary value of livestock and modern assets by multiplying the quantity of each asset by its estimated average price in Chinese Yuan based on generally available market data (Table S1). We summed asset values in their respective categories to create farm animal worth and modern asset value variables for each household. The sum of both asset values created a total inventoried assets variable for each household. Additionally, we calculated and aggregated mean values and Gini coefficients, with 95% confidence intervals calculated using 5,000 bootstrap replicates, for these three asset indices and monthly household income by village (*n* = 15; six matrilineal and nine patrilineal).

### Analysis

The first hypothesis is that increased participation in markets was associated with greater average income, which we measured at the household level. This was modeled using generalised linear models following a quasi-poisson functional form with a log link to account for overdispersion of positive, right-skewed outcome variables. There were three separate outcomes: household income, household modern asset value and household farm animal value. Predictor variables included distance to the primary tourism centre on Lugu Lake (in kilometres), which was measured at the village level and invariant across households within a given village; and dummy variables for the household's reported primary source of income (agriculture, business, gifts and remittances, property and rent, irregular salary, regular salary, tourism, and welfare and subsidy), with agriculture as the reference. The models of modern and farm animal asset value also included household income (in millions CNY). Cluster-robust standard errors were used to account for village-level clustering of households. These models were performed at the household level. Models were run with distance to tourism included as a squared variable (Table S2) to allow for the relationship between market participation and inequality to vary in a parabolic fashion. Results did not differ substantially in terms of model fitted from those presented in the main text, but suggest that a non-linear interpretation is possible.

The second hypothesis – that increased emphasis on material wealth leads to greater inequality – was tested at the village level. Given the small sample size, we used Gaussian family generalised linear models (identity link) with relatively few predictor variables. Predictor variables included the village mean values for each wealth variable, distance to the tourism centre and matrilineal vs. patrilineal kinship organisation. Models were run with mean wealth included as a squared variable (Table S3) to allow the relationship between wealth and inequality to vary in a parabolic fashion, as predicted by certain models (e.g. Kuznets) of inequality. Results did not differ in terms of model fit from those presented in the main text.

Finally, wealth by matrilineal vs. patrilineal kinship organisation interactions was included to test the third hypothesis – that matriliny should buffer against the effects of increasing wealth on inequality. Where the interaction term is not shown in a given model, it indicates that it did not improve model fit. It was not included in the second order models presented in the Supporting Information to avoid over-fitting. The root-mean-square errors (RMSE) were used for model comparison and standardised by dividing each RMSE by the maximum value of the outcome variable in the data for each model.

### Software

Data treatment and analysis were performed in R 4.1 (R Core Development Team, 2021); Gini coefficients were calculated using the ineq package (Zeileis et al., 2014, v0.2-13).

## Results

Our survey included usable data from 2332 individuals from 486 households in 15 villages (Table 1).

**Table 1.** Sample characteristics (*N* = 2332 individuals; 486 households; 15 villages)

Individual		
Age in years (mean, SD)	42.1	16.2
Women (*N*, %)	1177	50.47
Men (*N*, %)	1155	49.52
Household income in CNY last year (median, IQR)	32,500	15,000, 63,200
Household total inventoried asset value in CNY (median, IQR)	89,203	47,726, 129,855
Household modern asset value in CNY (median, IQR)	67,267	20,903, 83,613
Household farm animal worth in CNY (median, IQR)	24,876	14,570, 43,115
Village distance to Lugu Lake in km (mean, SD)		
Matrilineal area	12.05	6.39
Patrilineal area	72.67	7.41
Village Gini for household income (mean, SD)	0.41	0.08
Village Gini for total inventoried asset value (mean, SD)	0.31	0.05
Village Gini for modern asset value (mean, SD)	0.40	0.08
Village Gini for farm animal worth (mean, SD)	0.36	0.10

IQR, Interquartile range.

### Hypothesis 1 – Increasing market integration is associated with increased emphasis on material wealth

**Figure 1.**
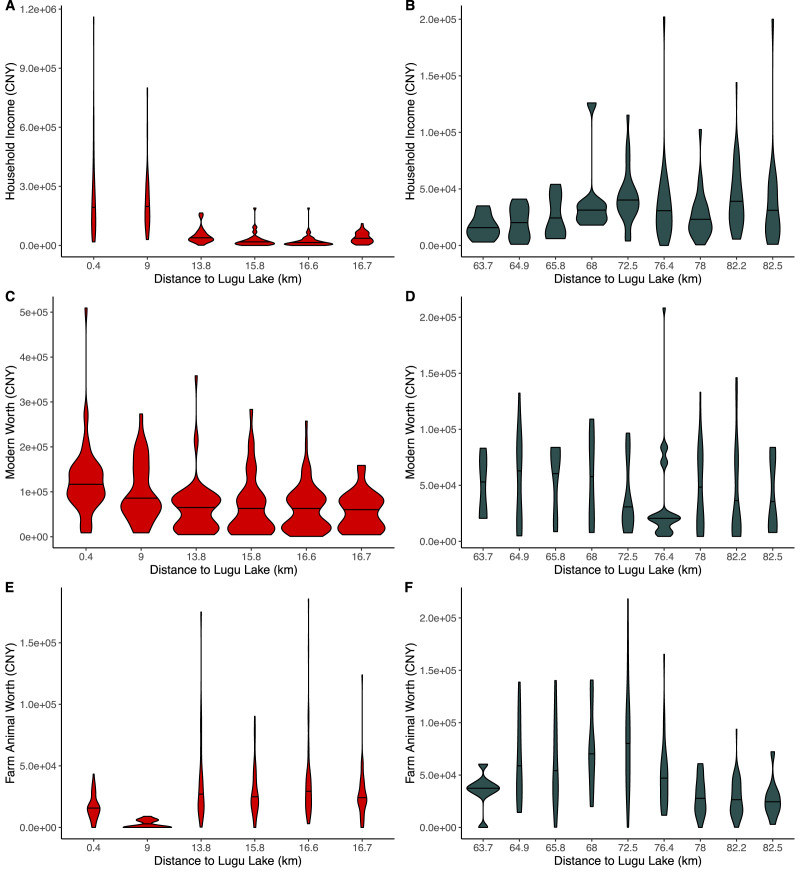
Violin plots showing the distribution of various types of material wealth by predominant lineality (matrilineal, red; patrilineal, gry) and distance to the primary tourism cwithin the area corresponding to each lineality. Each violin plot corresponds to a single village. (a, b) Househld income; (c, d) modern sset worth; (e, f) farm anial worth. Note that the *x*-axis is not to scale and there is a difference in scales on the *y*-axis.

In general, households near the primary tourism centre at Lugu Lake tended to have higher incomes and lower farm animal worth than households located farther away (Figure 1). The two closest villages differed significantly from all other villages in terms of income (Figure 1a, b) and farm animal worth (Figure 1e, f). Household modern asset worth (Figure 1c, d) did not appear to be as strongly differentiated. Wealth distribution across all three wealth types and most villages was highly non-normal with most households reporting comparatively low wealth and a few households reporting relatively high wealth. This trend seemed less apparent in patriliny (Figure 1b, d, f), where wealth distributions appeared more varied in shape. Again, the matrilineal villages closest to the lake stood out as having relatively even distributions of income and skewed distributions of farm animal worth, suggesting that more households had higher incomes there than in other locations. In other words, the high average income in the two matrilineal villages closest to the lake was attended by a relatively even distribution in income.

In matrilineal communities, village-level comparisons showed a similar pattern of declining material income/assets with increasing distance to the local tourism centre, particularly household income, with a marked difference after 10 km from the primary local site of tourism. Modern asset value was highest in the village closest to the centre of tourism (Figure 1c and d), whereas wealth in farm animals (Figure 1e and f) was greater in the villages farther from tourism, again, with a marked difference after 10 km. The same overall trends were apparent in patrilineal communities, although overall wealth was substantially lower and not clearly related to distance to tourism, consistent with higher reliance on agriculture among patrilineal Mosuo, who live in a more remote area distant from the Yongning basin and the major tourism draw of Lugu Lake.

Summary statistics indicated that income and modern asset wealth were higher near the lake and regression models generally supported this. Indeed, these models showed that income nearer to the lake was largely accounted for by differences in primary source of income. Distance to tourism was not significant in these models, because it was captured by the mix of activities that arise in association with market integration (Figure 3). Engagement with markets (primary sources of income from business, property, regular salary or tourism) was generally associated with increased income and modern asset wealth and decreased farm animal wealth (Table 2; see also Supporting Information, Fig. 3 and preceding text). Tests for overdispersion supported (*z* > 3.0, *p* < 0.001) the use of quasi-poisson models for all household level models. The RMSE analysis did not provide strong support for second order models over the first order models reported here: differences in RMSE were small, and qualitative conclusions were similar. Second-order models are presented in the Supporting Information for comparison. Fit was generally better in the models predicting income than the other assets.

**Table 2.** Generalised linear models (quasi-Poisson family, log link) of household income (top), modern asset value (middle) and farm animal worth (bottom). Three sets of models (overall, matrilineal and patrilineal) are presented for each outcome (household income, household modern asset value, household farm animal worth)

	Overall		Matrilineal		Patrilineal	
	Coefficient	95% CI	Coefficient	95% CI	Coefficient	95% CI
*Household income last year (CNY)*						
(Intercept)	10.38	(9.97, 10.80)	10.89	(9.62, 12.16)	9.887	(8.15, 11.62)
Distance to tourism center (per 10km)	−0.038	(−0.12, 0.04)	−0.44	(−1.21, 0.33)	0.047	(−0.17, 0.27)
*Primary source of household income (ref: agriculture)*						
Business	1.54	(0.76, 2.32)	1.742	(0.62, 2.86)	1.002	(0.64, 1.37)
Gifts/remittances	0.367	(0.073, 0.662)	0.407	(0.025, 0.788)	0.277	(0.088, 0.46)
Property and rent	1.913	(1.54, 2.29)	1.58	(0.36, 2.80)	−0.71	(−0.87, −0.55)
Salary (irregular)	0.182	(−0.076, 0.44)	0.074	(−0.22, 0.36)	0.231	(−0.049, 0.511)
Salary (regular)	0.613	(0.28, 0.94)	0.649	(0.26, 1.038)	0.73	(0.42, 1.037)
Tourism	1.796	(1.30, 2.29)	1.40	(−0.055, 2.86)		
Welfare/subsidy	−0.748	(−2.049, 0.55)			−0.93	(−2.34, 0.47)
RMSE	0.072		0.089		0.13	
*N*	467		273		194	
*Household modern asset value (CNY)*						
(Intercept)	11.0030	(10.88, 11.13)	11.14	(10.876, 11.40)	13.41	(12.88, 13.94)
Distance to tourism center (per 10km)	−0.064	(−0.083, −0.046)	−0.13	(−0.28, 0.030)	−0.41	(−0.49, −0.34)
*Primary source of household income (ref: agriculture)*						
Business	0.58	(0.26, 0.89)	0.34	(0.032, 0.66)	0.94	(0.32, 1.56)
Gifts/remittances	−0.046	(−0.53, 0.44)	−0.38	(−0.764, −0.0030)	0.74	(0.493, 0.98)
Property and rent	0.37	(0.13, 0.62)	0.26	(−0.048, 0.57)	0.21	(0.030, 0.39)
Salary (irregular)	0.074	(−0.091, 0.24)	0.068	(−0.14, 0.28)	0.28	(−0.004, 0.56)
Salary (regular)	0.394	(0.20, 0.58)	0.34	(0.19, 0.48)	0.59	(−0.103, 1.28)
Tourism	0.44	(0.17, 0.71)	0.32	(−0.042, 0.68)		
Welfare/subsidy	−1.74	(−2.27, −1.20)			−1.38	(−2.015, −0.74)
household income last year (per million CNY)	1.12	(0.73, 1.52)	1.15	(0.86, 1.45)	3.61	(−0.38, 7.60)
RMSE	0.010		0.12		0.16	
*N*	466		272		194	
*Household farm animal worth (CNY)*						
(Intercept)	10.57	(10.32, 10.81)	12.52	(11.96, 13.083)	12.87	(11.20, 14.55)
Distance to Tourism Center (per 10km)	0.043	(−0.010, 0.096)	−1.24	(−1.58, −0.90)	−0.28	(−0.50, −0.060)
*Primary source of household income (ref: agriculture)*						
Business	−0.85	(−1.20, −0.49)	−0.90	(−1.78, −0.0070)	−1.053	(−1.42, −0.69)
Gifts/remittances	−0.36	(−0.75, 0.026)	−0.10	(−0.36, 0.16)	−0.56	(−1.04, −0.092)
Property and rent	−1.46	(−2.44, −0.49)	−2.78	(−3.53, −2.027)	−0.11	(−0.35, 0.12)
Salary (irregular)	−0.49	(−0.79, −0.19)	−0.37	(−0.696, −0.037)	−0.46	(−0.871, −0.045)
Salary (regular)	−0.60	(−0.87, −0.34)	−0.45	(−0.60, −0.30)	−0.87	(−1.64, −0.097)
Tourism	−1.21	(−2.015, −0.40)	−2.63	(−3.29, −1.97)		
Welfare/subsidy	−2.91	(−4.44, −1.38)			−2.67	(−4.23, −1.12)
household income last year (per million CNY)	0.36	(−0.28, 1.00)	−0.94	(−1.24, −0.65)	4.71	(1.60, 7.82)
RMSE	0.13		0.13		0.14	
*N*	465		272		193	

As an example of the effects of income source on wealth, households that declared ‘business’ as their primary source of income had significantly higher incomes than households that declared ‘agriculture’, the reference category, as their main income source. At zero kilometres from the tourism centre, agricultural households are predicted to earn exp(10.38) = 32,275 CNY (~$4,500) annually; business-oriented households are predicted to earn exp(10.38 + 1.59) = 150,479 CNY (~$21,000) annually, with some households oriented towards business earning substantially higher sums.

### Hypothesis 2. An increased emphasis on material wealth is associated with increased inequality

**Figure 2.**
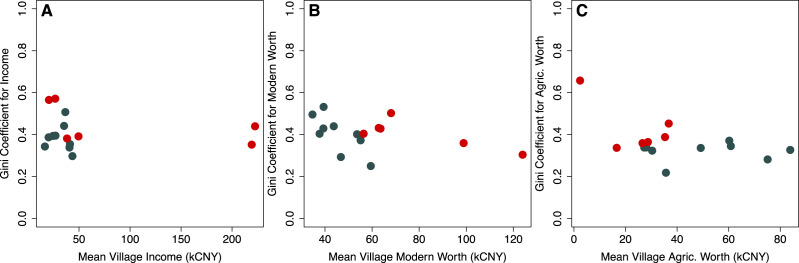
Village-level Gini coefficients for various forms of material wealth vs. the meue of the same form of material wealth for matriliny (red) and patriliny (grey): (A) househod incomeinequality; (b) modern asset ineqality; and (c) farm animal worth inequality.

We did not find support for this hypothesis. Figure 2 showed no trend towards higher Gini coefficients with increasing household income or wealth measures. In fact, the qualitative impression from Figure 2 was that the opposite may be more likely. Regression results also did not support an increase in inequality associated with increases in household income or modern asset value (Table 3). Indeed, the relationship between assets and Gini coefficient was often negative. Squared terms to allow for parabolic relationships between wealth and inequality (as measured by Gini coefficients) were incorporated into models in Supporting Information Table 2. Model fit was not improved by these terms.

**Table 3.** Generalised linear models (Gaussian family, identity link) of village Gini coefficients for household income (top), modern asset value (middle) and farm animal worth (bottom)

	Coefficient	95% CI
*Gini for household income*		
(Intercept)	0.42	0.36, 0.48
Village mean household income (per million CNY)	−0.14	−0.80, 0.51
RMSE: 0.14		
*Gini for modern assets*		
(Intercept)	0.49	0.38, 0.59
Village mean modern asset value (per million CNY)	−1.41	−3.00, 0.19
RMSE: 0.13		
*Gini for farm animal worth*		
(Intercept)	0.45	0.35, 0.54
Village mean farm animal worth (per million CNY)	−2.08	−4.15, 0.0030
RMSE: 0.12		

These inverse associations were generally more apparent when distance to Lugu Lake was included in models with income or asset predictors (Table 4). Wealth had more consistent, negative effects on Ginis than distance to lake.

**Table 4.** Generalised linear models (Gaussian family, identity link) of village Gini coefficients for household income (top), modern asset value (middle) and farm animal worth (bottom) including distance to the primary tourism location as a predictor

	Coefficient	95% CI
*Gini for household income*		
(Intercept)	0.51	0.40, 0.62
Village mean household income (per million CNY)	−0.53	−1.25, 0.19
Distance to Lugu Lake (tourist attraction)	−0.015	−0.030, −0.0010
RMSE: 0.12		
*Gini for modern assets*		
(Intercept)	0.63	0.39, 0.87
Village mean modern asset value (per million CNY)	−2.77	−5.35, −0.18
Distance to Lugu Lake (tourist attraction)	−0.013	−0.033 −0.0070
RMSE: 0.12		
*Gini for farm animal worth*		
(Intercept)	0.47	0.37, 0.56
Village mean farm asset value (per million CNY)	−1.17	−3.53, 1.20
Distance to Lugu Lake (tourist attraction)	−0.012	−0.029, 0.0050
RMSE: 0.12		

### Hypothesis 3. Kinship mediates relationships between inequality and wealth

Per capita household income was higher in the matrilineal area (which is much closer to the centre of tourism; *N* = 486, *t* = 5.4119, *p* = 0.0000, one-tailed *t*-test), as was inequality (*N* = 15, *t* = 1.4933, *p* = 0.0873). In regression analyses, mean village income and asset values remained inversely associated with Ginis with lineality included in models (Table 5). Within matrilineal communities, villages closer to the lake had lower Gini coefficients than those more removed from the lake (Figures 1 and 2). Patriliny was generally associated with lower Ginis. Interactions between lineality and asset value were apparent for modern and farm assets. Mean modern asset value was inversely associated with inequality in modern assets in both matriliny and patriliny, but this association was stronger in patriliny.

**Table 5.** Generalised linear models (Gaussian family, identity link) of village Gini coefficients for household income (top), modern asset value (middle), and farm animal worth (bottom) including lineality as a predictor. The *p*-value for the coefficient on the interaction term in the modern asset and farm animal worth models was <0.10 and is included

	Coefficient	95% CI
*Gini for household income*		
(Intercept)	0.50	0.41, 0.58
Village mean household income (per million CNY)	−0.50	−1.16, 0.15
Patriliny (ref: matriliny)	−0.099	−0.18, −0.012
RMSE: 0.115		
*Gini for modern assets*		
(Intercept)	0.57	0.41, 0.72
Village mean modern asset value (per million CNY)	−2.052	−3.96, −0.14
Patriliny (ref: matriliny)	0.18	−0.088, 0.44
Mean modern asset value × patriliny	−5.46	−10.44, −0.48
RMSE: 0.093		
*Gini for farm animal worth*		
(Intercept)	0.57	0.43, 0.70
Village mean farm animal value (per million CNY)	−5.89	−10.78, −0.86
Patriliny (ref: matriliny)	−0.26	−0.44, −0.070
Mean farm asset value × patriliny	5.99	0.48, 11.50
RMSE: 0.096		

For farm assets, the inverse association between farm asset value and inequality in farm animal worth in matriliny was unapparent in patriliny. Because agriculture is so much more important to livelihoods in patrilineal areas than matrilineal ones, this distinction is probably meaningful. Higher mean asset value was consistently associated with lower inequality in matrilineal areas. In patrilineal areas, that was the case for modern assets, but not for farm animal worth.

All told, our data painted a picture of greater participation in markets increasing household incomes and modern assets. However, we did not find evidence that incomes and assets increased inequality. Indeed, our data suggested that matriliny was more unequal than patriliny and that, within each area – matrilineal and patrilineal – villages with higher average income or asset value were *more*, rather than less, equal. The only exception to this pattern was farm animal worth in the patrilineal area, which was positively associated with inequality.

**Figure 3.**
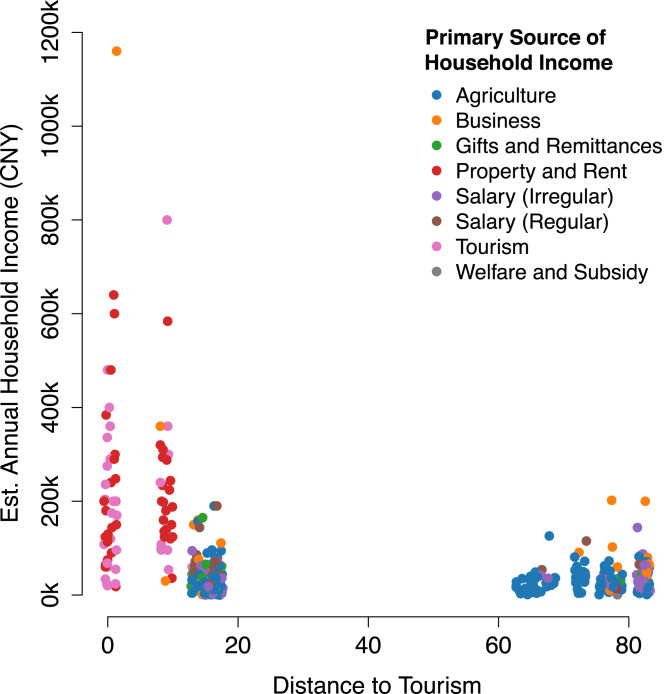
The relationship beween a household's distance to the tourism centre (*x*-axis), estiannual household income (CNY, *y*-axis) and primary source of houshold income (marker colour); a small amount of noise is applied to the *x*-values to reduce overplotting.

Figure 3 displays graphically a potential reason why distance from the tourism centre was not a significant predictor of household income in Table 2: the two villages closest to the tourism centre had a very different mix of income sources than villages farther away, appearing almost completely reliant on market activity whereas villages farther away were predominantly agricultural. In other words, ‘inequality’ as measured by Gini indices far from the main source of tourism may represent a mix of subsistence strategies rather than true differentiation on some global measure of wealth. Inequality in farm assets in patrilineal areas also corresponds to a stronger apparent emphasis on agriculture as a means of subsistence.

## Discussion

In this paper, we set out to investigate patterns of inequality, market integration and kinship systems to see if they conformed to the hypothesis that matriliny is less unequal than patriliny. This hypothesis is based on both sociological insights that note more redistributive norms among matrilineal communities (e.g. Dyson & Moore, 1983; see Mattison, 2016; Mattison, Sum et al., 2021) and theoretical arguments from behavioural ecology that establish the benefits of patriliny for the defence of wealth (Alvard, 2003) and inequalities associated with son-biased parental investment (Sieff, 1990), female emigration (Bentley et al., 2012), male economic strategies (e.g. Alesina et al., 2013) and unigeniture (see Hrdy & Judge, 1993). To investigate evidence relevant to this hypothesis, we set up a comparison of intensification of reliance on material wealth (Borgerhoff Mulder et al., 2009; Bowles et al., 2010) owing to market integration (Lu, 2007; Mattison, Hare et al., 2021) in communities of Mosuo, both matrilineal and patrilineal, experiencing rapid market integration associated primarily with tourism (see also Mattison, Sum et al., 2021; Walsh, 2005). We tested each step in this argument, establishing first that market integration leads to some sort of intensification of wealth, but much more clearly in matrilineal communities and in particular those abutting the lake, where tourism is strongest. Next, we asked whether intensified reliance on material wealth, especially as reflected in income and modern assets, was associated with increased inequality. The answer here, overall, is no – in fact, like others (Godoy et al., 2004; Gurven et al., 2015), we generally found *negative* relationships where higher wealth or market integration was associated with less inequality. The one exception to this was in relation to farm animal worth in patriliny, where increased farm animal value was associated with increased inequality. This may be important, given a heavy reliance on agriculture in patriliny, and therefore perhaps increased motivation to amass related resources, but otherwise the differences based on kinship are slight.

Market integration is a powerful force of economic, health and lifestyle change (e.g. Colleran, 2020; Godoy, Byron et al., 2005; Godoy et al., 2004; Gurven et al., 2015; Lu, 2007; Mattison, Sum et al., 2021; Wu, 2019). It is not, however, a linear process (e.g. see Figure 1); its outcomes are sensitive to local context (Godoy, Reyes-Garcia et al., 2005), including starting points (e.g. different phases of market integration are expected to yield different outcomes), opportunity structures and the cultural norms and institutions shaping responses to those opportunities (see Currie et al., 2021). Our results demonstrate clearly that market integration in response to tourism involves a shift from subsistence- to market-oriented activities, consistent with Lu's definition of market integration (Lu, 2007), but they do not map cleanly onto wealth or inequality. For example, farm animals were more plentiful in villages more remote from tourism whereas ‘modern’ assets and household income were highest in villages closer to tourism (Figure 1), indicating a shift towards a more market integrated economy. This pattern is more obvious in matrilineal communities, where tourism has been a source of income for nearly 40 years (e.g. Luo, 2008; Mattison, 2010; Walsh, 2001) and only nascent in patrilinal communities, where tourism has a more marginal impact on the local economy. Distance to tourism, however, does not appear to exert a strong influence on wealth inequality (see also Godoy et al., 2004; Gurven et al., 2015). Rather, income and assets, per se, have more consistent, negative effects on inequality as captured by Gini coefficients (Table 4).

Contrary to our predictions, overall, the level of inequality was substantially higher in matriliny than in patriliny. This is true across categories of material wealth, with the smallest differences seen in farm animal worth and the largest values seen in household income (see also Tucker et al., 2015). The levels of inequality are also not trivial. The highest inequalities in household income arise among matrilineal villages; approaching 0.60, these Gini coefficients are on par with those of the most unequal nation states (e.g. South Africa) and exceeding those of other small-scale pastoral and agricultural societies (ca. 0.4–0.5; Smith, Borgerhoff Mulder et al., 2010). Gini coefficients in farm animal worth were relatively small in both matriliny and patriliny; at an average of 0.35, this is more similar to, e.g. the UK, and on par with other pastoral and agricultural societies. At the same time, the results may offer tentative, descriptive evidence of matrilineal redistributive norms in action. Within the matrilineal area, Gini coefficients declined with average village wealth. Redistributive norms in matriliny are supported by participants’ attestations that there are intentional schemes involving communal management of income from tourism to avoid wealth monopolisation. For example, villages require at least one individual from every household to participate in group activities, such as dances and boat riding, and income from these activities is then split equally among households. This means that even households that are otherwise reliant on agriculture still receive regular income from village-level tourism activities. Relatively low Gini coefficients despite high wealth are also apparent in modern assets, presumably subject to less regulation (although still based on income), whereas there is limited differentiation in village inequality based on farm animal value.

Overall, our results offer additional evidence that market integration does not act in a directed way to increase wealth or inequality and that its effects are highly dependent on existing social norms and institutions, including those related to kinship. Gurven and colleagues (2015) also found that market integration did not alter traditional sharing networks among Bolivian forager–horticulturalists and did not increase inequality. On the contrary, sharing depth was increased in villages that exhibited higher market integration and higher wealth. They speculated that this was due to increased income being used for signalling purposes rather than amassed for personal gain. This is consistent with other studies highlighting redistribution of wealth owing to demand sharing among kin (e.g. Stack, 1974), and the difficulty of personal accumulation without extracting oneself from local networks or tipping points being reached that inspire individualistic autonomy among a sufficient proportion of a population (Haagsma & Mouche, 2013; Lesorogol, 2003). At the same time, it is clear that both in China (e.g. Wu, 2019) and elsewhere (Ensminger, 1996; Lesorogol, 2003; Putsche, 2000; Yellen, 1990), market integration, globalisation and privatisation often eventually lead to greater autonomy and increased potential for inequality. Given the distinct advantages of inherited wealth in such contexts (Gudeman, 2015; Piketty, 2015; Yanagisako, 2015), kinship should not be ignored in investigations of these dynamics. We speculate that as market integration proceeds, patrilineal communities will have more difficulty evading inequality given pre-existing biases in inheritance, higher fertility (Mattison, Beheim et al., 2016; Shih & Jenike, 2002; see also Leonetti et al., 2007; Gudeman, 2015) and greater circumscription of habitable terrain. More generally, kinship systems reflect gendered cooperation and conflict (Leonetti et al., 2007) and the dynamics that result when families organise differentially around gender to support their needs (Mattison, Shenk et al., 2019). Gendered kinship systems are known to impact health (Mattison, MacLaren et al., 2021) and may, as tentatively suggested here, have implications for the escalation of inequality and any concomitant effects on health. Gendered conflict must, therefore, be studied at multiple levels of manifestation, from within households to across communities and populations, to understand its variable effects on health and livelihoods.

The study is subject to a number of limitations. First, Gini coefficients are highly sensitive to outliers, so inclusion or exclusion of a single data point in small samples can make a big difference. We took every precaution in our inspections of raw data to mitigate this limitation, but it remains the case that, in the initial phases of wealth acquisition, in instances where individual households become wealthy earlier than others, Gini coefficients are likely to be inflated and unstable, and to over-represent the extent of inequality in mixed economies (Tucker et al., 2015). Indeed, the meaning of inequality as captured in metrices like the Gini coefficient is probably less consistent in mixed economies, where people rely on a variety of ways to make ends meet (Baird & Gray, 2014; BurnSilver et al., 2016; Hackman & Kramer, 2021) than it is in economies where people rely purely on income. Investigating the changes in Gini coefficients in the same locations over time and over a multitude of wealth types will help in efforts to establish causality and establish patterns in volatile spaces. Secondly, and more importantly, this case study would be a stronger comparison if the patrilineal and matrilineal communities were in similar phases of economic transition – it is clear in these data that the patrilineal communities remain much more oriented towards agricultural activities than some of the matrilineal communities, which have long been oriented towards tourism. At the same time, the source of wealth intensification (e.g. tourism vs. government initiatives vs. business) does not seem to play a strong role in driving the relationship between wealth and inequality. Thus, comparative studies that involve communities with variation in kinship and gender norms as well as market integration variables will also be suitable for future study. Finally, there is no consideration of debt in our assessment of wealth. We expect this would decrease the levels of inequality further in locations dedicated to tourism; however, given that these are also the most likely areas to succumb to debt (e.g. to construct guest houses), including measures of debt would probably have reinforced findings.

## Conclusion

Our study shows that market integration leads to changes in wealth and livelihoods, but that these are not easily mapped onto changes in inequality. While long-run dynamics may eventually stabilise and follow our predictions, the study reinforces the importance of ethnographic detail in understanding local deviations from anticipated patterns (Gurven et al., 2015; Peebles, 2015; Tucker et al., 2015). This will be crucial within a context where inequality is rising sharply (Wu, 2019) and more generally where aggregate statistics obscure important local dynamics that have greater potential to illustrate causality (see Pollet et al., 2014). This is particularly important in studies of inequality, which is likely to be most effectively reduced or exacerbated by institutions (Scheffer et al., 2017), including those based on kinship (Yanagisako, 2015). Although the effects of kinship were not easily seen here, we suspect strongly that they will become more apparent as the patrilineal communities become more economically developed. The gender dynamics inherent to these systems will also have important implications for things that are plausibly downstream of inequality, such as health (Jaeggi et al., 2021; Reynolds et al., 2020), and contribute to recent calls to consider inequality and justice in terms of drivers that move beyond straightforward economic indicators (Mao et al., 2022).

## Research transparency and availability

Data analysed for this manuscript are available in Github at https://github.com/ngmaclaren/mosuo-inequality-ehs. Certain data included in these analyses (location-based data) are only available via request to protect the anonymity of participants.

## Supplementary material

For supplementary material accompanying this paper visit https://doi.org/10.1017/ehs.2022.52. click here to view supplementary material
