# Supplementary material for: Market integration, income inequality, and kinship system among the Mosuo of China
Source: Evol Hum Sci. 2022 Nov 21;5:e4. doi: 10.1017/ehs.2022.52 (PMC10426023; doi:10.1017/ehs.2022.52)
Supplement: Supplementary file 1 [file S2513843X22000524sup001.docx]

Supplemental Information – Market integration, income inequality, and kinship system among the Mosuo of China

Table S1. List of farm animal and modern assets, their approximate values in Yuan (CNY), and the sources we used to assign those values. These values were used to calculate farm animal and modern asset worth indices for plots and regression analysis.

| **Asset** | **Average Value (CNY)** | **Index** | **Source** |
| --- | --- | --- | --- |
| *Bovidae* | 2,500 | Farm Animal | https://www.foodingredientsfirst.com/news/rabobank-highlights-record-prices-in-beef-market-strong-demand-in-china-observed.html |
| *Caprinae* | 2,320 | Farm Animal | https://www.usmef.org/chinas-sheep-meat-market-weak-due-to-increased-domestic-and-foreign-supplies/ |
| *Equidae* | 2,560 | Farm Animal | Average value of ungulates above |
| Pigs | 2,860 | Farm Animal | https://www.pig333.com/markets_and_prices/china_106/ and average weight of domestic pig (1450-300 kg) |
| Poultry | 18 | Farm Animal | https://www.selinawamucii.com/insights/prices/china/live-chicken/ and using average weight of 2.kg |
| Air Conditioner | 3,000 | Modern | https://www.coolingpost.com/world-news/ac-prices-expected-to-rise-as-china-gets-smart/ |
| Car | 62,200 | Modern | https://www.statista.com/statistics/875078/average-price-of-used-cars-sold-in-china/ |
| Cell Phone | 637 | Modern | https://www.quora.com/What-is-the-cost-of-a-smartphone-in-China |
| Motorcycle | 13,000 | Modern | Online market sites (e.g., alibaba.com) |
| Refrigerator | 3,200 | Modern | American appliance retail sites (least expensive models) |
| Television | 3,748 | Modern | https://www.globaltimes.cn/page/202103/1217379.shtml |
| Washing Machine | 318 | Modern | Based on semi-portable designs on alibaba.com |

### Second Order Models

Table S2: Second Order Market Participation Model. Note that the primary coefficient for distance to tourism is of different magnitude here compared to the main text Table 2 due to the polynomial fit.

|  |  |  | Overall | Matrilineal | | | Patrilineal | | |
| --- | --- | --- | --- | --- | --- | --- | --- | --- | --- |
|  |  | 95% CI | |  | 95% CI | |  | 95% CI | |
| Household income last year (CNY) | Coef | Lower | Upper | Coef | Lower | Upper | Coef | Lower | Upper |
| (Intercept) | 10.34 | 10.08 | 10.6 | -17.29 | -36.38 | 1.81 | 1.01 | -8.61 | 10.64 |
| Distance to Tourism Center | -3.593 | -9.335 | 2.148 | -763.227 | -1287.241 | -239.213 | 170.687 | -8.073 | 349.446 |
| (Distance to Tourism Center)^2^ | 3.870 | -2.240 | 9.979 | -121.821 | -205.594 | -38.049 | -35.569 | -74.095 | 2.958 |
| Primary source of HH income (Ref: Agriculture) |  |  |  |  |  |  |  |  |  |
| Business | 1.394 | 0.731 | 2.057 | 0.975 | 0.535 | 1.414 | 1.042 | 0.613 | 1.471 |
| Gifts/Remittances | 0.279 | -0.029 | 0.586 | 0.137 | -0.123 | 0.397 | 0.349 | 0.140 | 0.558 |
| Property and rent | 1.529 | 0.537 | 2.522 | 0.408 | -0.735 | 1.551 | -0.793 | -0.972 | -0.614 |
| Salary (irregular) | 0.101 | -0.166 | 0.367 | -0.122 | -0.396 | 0.152 | 0.293 | 0.046 | 0.540 |
| Salary (regular) | 0.573 | 0.256 | 0.889 | 0.482 | 0.153 | 0.812 | 0.779 | 0.405 | 1.153 |
| Tourism | 1.367 | 0.149 | 2.585 | 0.272 | -1.086 | 1.631 |  |  |  |
| Welfare/Subsidy | -0.901 | -2.191 | 0.390 |  |  |  | -0.920 | -2.397 | 0.558 |
| RMSE | 0.071 |  |  | 0.085 |  |  | 0.127 |  |  |
| N | 467 |  |  | 273 |  |  | 194 |  |  |
|  |  |  |  |  |  |  |  |  |  |
|  |  |  | Overall | Matrilineal | | | Patrilineal | | |
|  |  | 95% CI | |  | 95% CI | |  | 95% CI | |
| Household modern asset value (CNY) | Coef | Lower | Upper | Coef | Lower | Upper | Coef | Lower | Upper |
| (Intercept) | 10.73 | 10.6 | 10.85 | 15.75 | 10.41 | 21.1 | 14.31 | 9.51 | 19.1 |
| Distance to Tourism Center | -4.149 | -5.465 | -2.834 | 131.960 | -15.845 | 279.765 | -74.627 | -165.051 | 15.797 |
| (Distance to Tourism Center)^2^ | -1.177 | -3.423 | 1.070 | 23.704 | -0.519 | 47.926 | 9.898 | -9.474 | 29.271 |
| Primary source of HH income (Ref: Agriculture) |  |  |  |  |  |  |  |  |  |
| Business | 0.617 | 0.269 | 0.965 | 0.460 | 0.257 | 0.664 | 0.917 | 0.285 | 1.550 |
| Gifts/Remittances | -0.020 | -0.530 | 0.490 | -0.334 | -0.723 | 0.055 | 0.709 | 0.461 | 0.957 |
| Property and rent | 0.479 | 0.090 | 0.868 | 0.460 | 0.129 | 0.790 | 0.236 | 0.038 | 0.434 |
| Salary (irregular) | 0.098 | -0.071 | 0.266 | 0.099 | -0.153 | 0.351 | 0.259 | -0.043 | 0.560 |
| Salary (regular) | 0.406 | 0.206 | 0.607 | 0.363 | 0.180 | 0.547 | 0.570 | -0.137 | 1.278 |
| Tourism | 0.557 | 0.128 | 0.987 | 0.505 | 0.124 | 0.886 |  |  |  |
| Welfare/Subsidy | -1.691 | -2.258 | -1.124 |  |  |  | -1.380 | -1.994 | -0.767 |
| HH income last year (million CNY) | 1.188 | 0.808 | 1.569 | 1.207 | 0.895 | 1.519 | 3.812 | -0.549 | 8.174 |
| RMSE | 0.100 |  |  | 0.116 |  |  | 0.161 |  |  |
| N | 466 |  |  | 272 |  |  | 194 |  |  |
|  |  |  |  |  |  |  |  |  |  |
|  |  |  | Overall | Matrilineal | | | Patrilineal | | |
|  |  | 95% CI | |  | 95% CI | |  | 95% CI | |
| Household farm animal worth (CNY) | Coef | Lower | Upper | Coef | Lower | Upper | Coef | Lower | Upper |
| (Intercept) | 10.6 | 10.4 | 10.8 | 14.53 | 3.6 | 25.46 | -1.19 | -8.96 | 6.58 |
| Distance to Tourism Center | 4.488 | 0.686 | 8.289 | 95.324 | -204.674 | 395.321 | 220.890 | 75.550 | 366.230 |
| (Distance to Tourism Center)^2^ | -3.978 | -8.226 | 0.271 | 33.981 | -20.622 | 88.584 | -52.804 | -83.621 | -21.987 |
| Primary source of HH income (Ref: Agriculture) |  |  |  |  |  |  |  |  |  |
| Business | -0.711 | -1.039 | -0.383 | -0.817 | -1.476 | -0.159 | -0.859 | -1.294 | -0.423 |
| Gifts/Remittances | -0.241 | -0.548 | 0.066 | -0.054 | -0.282 | 0.173 | -0.366 | -0.850 | 0.118 |
| Property and rent | -1.099 | -2.305 | 0.107 | -2.877 | -3.459 | -2.295 | -0.204 | -0.472 | 0.064 |
| Salary (irregular) | -0.388 | -0.659 | -0.117 | -0.337 | -0.694 | 0.020 | -0.305 | -0.664 | 0.055 |
| Salary (regular) | -0.542 | -0.766 | -0.317 | -0.422 | -0.593 | -0.251 | -0.731 | -1.416 | -0.045 |
| Tourism | -0.789 | -1.833 | 0.255 | -2.763 | -3.315 | -2.212 |  |  |  |
| Welfare/Subsidy | -2.759 | -4.362 | -1.156 |  |  |  | -2.616 | -4.303 | -0.929 |
| HH income last year (million CNY) | 0.574 | -0.232 | 1.380 | -1.066 | -1.410 | -0.722 | 3.199 | 0.386 | 6.012 |
| RMSE | 0.127 |  |  | 0.127 |  |  | 0.138 |  |  |
| N | 465 |  |  | 272 |  |  | 193 |  |  |

Table S3: Second Order Inequality Model

| Income, 2nd Order | Coef | Lower | Upper |
| --- | --- | --- | --- |
| (Intercept) | 0.411 | 0.369 | 0.453 |
| Mean HH income | -0.036 | -0.198 | 0.127 |
| (Mean HH income)^2^ | 0.094 | -0.068 | 0.256 |
| RMSE | 0.130 |  |  |
|  |  |  |  |
| Modern Worth, 2nd Order |  |  |  |
| (Intercept) | 0.404 | 0.365 | 0.442 |
| Mean HH modern worth | -0.127 | -0.277 | 0.023 |
| (Mean HH modern worth)^2^ | 0.017 | -0.133 | 0.167 |
| RMSE | 0.128 |  |  |
|  |  |  |  |
| (Intercept) | 0.363 | 0.325 | 0.401 |
| Mean HH farm animal worth | -0.171 | -0.318 | -0.025 |
| (Mean HH farm animal worth)^2^ | 0.180 | 0.034 | 0.327 |
| RMSE | 0.102 |  |  |

### Demonstration of Model Fit for Market Integration Models

The market integration models presented in the main text have coefficients that at times appear to have counter-intuitive directions. The two figures below demonstrate the effect of separating the models by lineality for the modern asset and farm animal value models.

Fig S1: Modern Asset Value


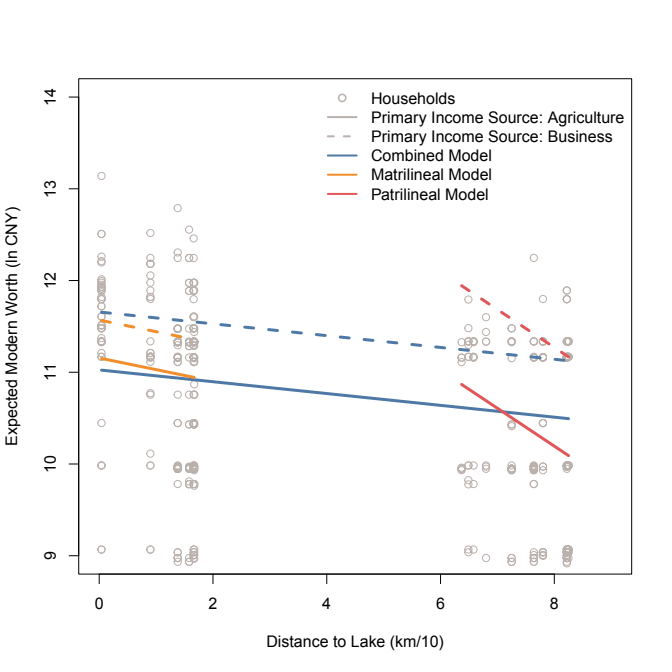


This figure shows the data as it is transformed to estimate the quasi-poisson models in the main text, with distance (km/10) on the x-axis and modern worth (ln CNY) on the y-axis. The data for each household is represented by a gray circle. Models are separated by color, with the combined model shown in blue, the matrilineal model shown in yellow, and the patrilineal model shown in red. Two levels of the qualitative predictor variable, primary income source, are shown as well: agriculture, the reference, is shown by a solid line, and business is shown by a dashed line. The relevant median income (in million CNY) was used in all cases: predicted values for the combined model used the sample median, whereas the models for the matrilineal and patrilineal areas used the matrilineal or patrilineal household median income.

In the modern asset value models, the patrilineal model has a much higher intercept than the other models. However, small distance values are not meaningful for this model: all patrilineal households are more than 60 kilometers from the lake. As can be seen in Fig. S1, once appropriate values for distance are entered into the model, the model produces reasonable predicted values. The dashed line does not pass through many data points because there are few households that declared business as their primary source of income.

Figure S2


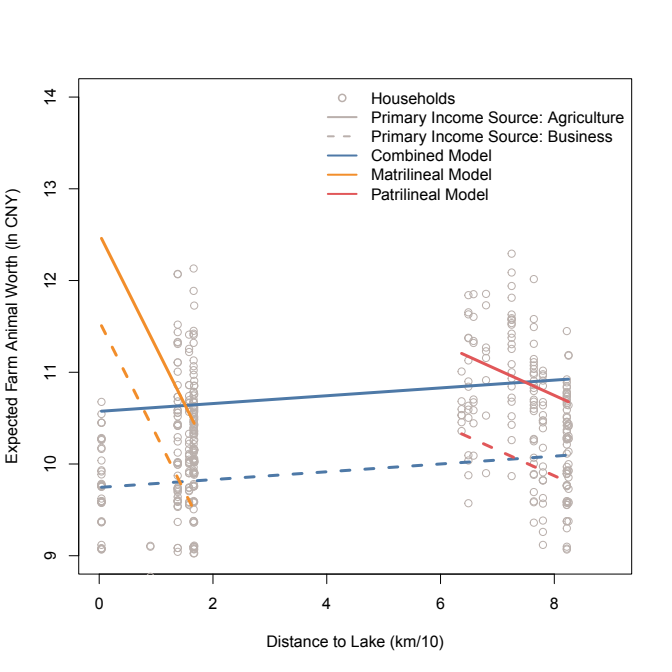


Fig. S2 shows the farm animal worth models; symbols and colors are the same as for Fig. S1. In this model, the confidence interval for the coefficient on distance in the combined model includes zero: taking all the data together, distance from the lake is not a meaningful predictor of household farm animal worth. However, the slope within each area is negative, and the confidence interval does not include zero.

Zooming in on the matrilineal case (Fig. S3), we can see that, if income *source* is not included in the model, distance to the lake is associated with increased farm animal worth, as expected. Adding the income source complicates the story: All income sources are associated with a decline in farm animal worth relative to agriculture; however, with source of income and household income controlled, distance to the lake then has a significantly negative association with farm animal worth. This negative association means that, for all households with a given source of income and income level, distance to the lake decreases farm animal worth. There are many reasons that could explain this, including relatively limited access to animal markets, but these are highly speculative given the evidence we have on hand. Overall, however, it is clear that increased proximity to the lake is associated with shifts toward more market-oriented lifestyles, increased income, and decreased farm animal worth.
